# β-patchoulene alleviates cognitive dysfunction in a mouse model of sepsis associated encephalopathy by inhibition of microglia activation through Sirt1/Nrf2 signaling pathway

**DOI:** 10.1371/journal.pone.0279964

**Published:** 2023-01-06

**Authors:** Ye Tian, Lin Wang, Xiaojing Fan, Hui Zhang, Zhiwei Dong, Tianzhu Tao

**Affiliations:** 1 Department of Anesthesiology, Sixth Medical Center of Chinese PLA General Hospital, Beijing, China; 2 Department of General Surgery, Air Force Medical Center, Beijing, China; 3 Department of Anesthesiology, Air Force Medical Center, Beijing, China; 4 Department of Neurosurgery, Air Force Medical Center, Beijing, China; Sungkyunkwan University and Samsung Advanced Institute of Health Science and Technology (SAIHST), REPUBLIC OF KOREA

## Abstract

**Background:**

Sepsis associated encephalopathy (SAE) is a common but poorly understood complication during sepsis. Currently, there are no preventive or therapeutic agents available for this neurological disorder. The present study was designed to determine the potential protective effects of β-patchoulene (β-PAE) in a mouse model of SAE and explore the putative mechanisms underpinning the beneficial effects.

**Materials and methods:**

SAE was induced in C57BL/6 mice by cecal ligation and puncture(CLP). Mice were administrated with β-PAE or saline by intra-cerebral ventricle(*i*.*c*.*v*) injection immediately after CLP surgery. The inhibitory avoidance tests and open field tests were performed at 24h, 48h and 7days after procedures. Cytokines expression, oxidative parameters, microglia polarization and apoptosis in the brain tissue were assessed. Sirt1, Nrf2, HO-1and cleaved-caspase3 expression in hippocampus was determined by western-blotting. Further, serum cytokines expression and spleen lymphocytes apoptosis were evaluated, and survival study was performed.

**Results:**

Septic mice suffered severe cognitive decline following CLP as evidenced by decreased memory latency time and lower frequency of line crossing in the behavioral tests. A high dose of β-PAE(1mg/kg) improved the cognitive impairment in SAE mice, which was accompanied by reduced cytokines expression and oxidative stress. Immunofluorescence assay showed that β-PAE inhibited the expression of Iba-1 and iNOS in microglia. The mechanistic study indicated that β-PAE could promote the nuclear expression of Sirt1/Nrf2 and enhance cytoplasmic HO-1 expression. Furthermore, *i*.*c*.*v* administration of β-PAE decreased the expression of serum cytokines and apoptosis in the spleen, thus leading to an improved 7-day survival of septic mice. Finally, blockade of Nrf2 activation with ML385 largely mitigated the protective effects of β-PAE on the cognitive function, neuroinflammation and survival in SAE mice.

**Conclusion:**

In this study, we found that β-PAE significantly altered sepsis induced neuroinflammation and microglia activation, thus reversed the cognitive decline and improved the peripheral immune function. The neuroprotective effects were possibly mediated by the activation of Sirt1/Nrf2/HO-1 pathway. β-PAE might serve as a promising therapeutic agent for SAE prevention and treatment.

## 1 Introduction

Sepsis is life-threatening organ dysfunction caused by a dysregulated host response to infection, impacting millions of people worldwide each year [1]. Sepsis associated encephalopathy(SAE) is among the most common but poorly understood complications of sepsis [2]. Approximately 70% of septic patients were observed with neurological symptoms ranged from confusion to delirium and, in worse cases, even coma [3]. It has been identified that SAE is strongly associated with higher mortality and long-term cognitive dysfunction [4,5]. Despite steady progress has been achieved in the understanding of the pathogenesis of SAE, no curative or preventive strategies are currently available, implying that the precise knowledge of immunopathological mechanisms remains far to be elucidated.

The pathophysiology of SAE is multifactorial, comprising neuroinflammation, neurotransmitter dysfunction, ischemic lesions, blood brain barrier(BBB) dysfunction and microglia activation [6]. Microglia plays a crucial role in orchestrating the immune response in the central nervous system(CNS) during sepsis. Upon stimulation by bacterial products or cytokines, microglial cells activate and present morphological, immunological and metabolic alterations [7,8]. Activated microglial cells are very plastic and may exist in multiple phenotypes, ranging from pro-inflammatory(M1) cell population releasing nitric oxide, TNF-α and IL-1β to anti-inflammatory subtype releasing IL-10 or IL-4. The proinflammatory phenotypes are usually considered to be neurotoxic whereas M2 phenotype may contribute to neuroprotection [9,10]. Consistently, the inhibition of M1 polarization by an intracerebroventicular(*i*.*c*.*v*) injection of minocycline suppressed the oxidative damage, neuroinflammation and long-term cognitive impairments in septic rats [11].Thus, modulation of microglia activation may provide with a promising approach in treating SAE.

β-patchoulene(β-PAE, C_15_H_24_), one of the major active ingredient of *Pogostemon cablin*, possesses potent anti-oxidative and anti-inflammatory properties [12,13]. Zhang *et al* reported that pretreatment with β-PAE exhibited a neuroprotective effect on cerebral I/R injury in rats via inactivating the TLR4/NF-ƘB signaling pathway [14]. Furthermore, we recently found that β-PAE preconditioning protects mice against hepatic I/R, which was at least in part through the suppression of M1 polarization of Kupffer cells and hepatocellular apoptosis [15]. These results rendered β-PAE a promising therapeutic agent in SAE which was implicated in the oxidative stress, neuroinflammation and microglia activation. Thus, this study was designed to determine the potential effects of β-PAE on the microglial activation and cognitive function in a mouse model of SAE.

## 2. Materials and methods

### 2.1 Animals and models of SAE

Male C57BL/6 mice aged 6–10 week were obtained from SPF(Beijing)Biotechnology Co.,Ltd. The study protocol was approved by the Institutional Animal Care and Use Committee of Air Force Medical Center (NO.20210317V1).

A mouse model of SAE was induced by cecal ligation and puncture (CLP). After one week of adaptive feeding, mice were randomly divided into CLP or sham group. Briefly, mice were anesthetized with inhaled isoflurane (induced at 3%, and maintained at 2% in 1-2L/min O_2_ throughout procedures), and then a middle line incision was made. Cecal was exposed, ligated at 1cm distally, and pierced through and through with a 22G needle. Sham-operated mice received cecal exposure, but without ligation or puncture. The incision site was infiltrated with 0.5ml 1% lidocaine. Each mouse was subcutaneously injected with 1ml lactated Ringer’s solution after surgery. Mice had free access to food and water during the experimental period.

### 2.2 Ethical statement

All animals were checked twice per day by trained professionals to identify deteriorating mice and prevent them from suffering. Mice were euthanized if they were moribund, immobile, unable to consume food and/or water, manifested with prolonged diarrhea, significantly labored breathing, post-surgical wound dehiscence or visible infection. Body temperature, fur appearance, posture, mobility, alertness, startle and righting reflex were monitored starting with 6h post-CLP. All mice were premedicated with buprenorphine(0.05 mg/kg) for postoperative analgesia.

### 2.3 Reagents and treatment

β-PAE was obtained from patchouli oil with a purity over 98% as described [12]. Anti-Nrf2 ab (clone103, MABE1799), anti-HO-1 ab (cloneHO-1-1, 374087), anti-Histone H3 ab(H0164), anti-GAPDH ab(G9545), and ML385 were purchased from Sigma-Aldrich (St.Louis, MO, US). Anti-iNOS ab(D6B6S), anti-Sirt1 ab and the secondary antibodies (Goat anti-rat CY3-conjugated IgG, Goat anti-rabbit FITC-conjugated IgG) were purchased from Cell Signaling Technology (MA, USA). Anti-CD11b ab and anti-Iba-1 ab were obtained from Abcam (Cambridge, UK).

β-PAE or ML385 was prepared in 3μL according to the designated dose. Mice were *i*.*c*.*v* injected with β-PAE immediately following CLP, and then treated with or without ML385 at 2 hours post procedures. For the control purpose, mice in control groups received equal volume of saline. A stereotactic instrument was used to ensure the accuracy and reliability.

### 2.4 Behavioral tests

#### 2.4.1 Step down inhibitory avoidance test

Mice were kept in a 50*25*25cm box with parallel stainless teel bars spaced 1cm apart and a platform(5*2.5cm) placed on the floor. During the training session, mice were placed on the platform and stimulated with a 0.4mA 2s shock immediately after stepping on the grid. The retention test was identical to the training session but without the electronic stimulation. The step-down latency time (maximum 180s) was recorded by an automatic device and used to assess the inhibitory avoidance memory.

#### 2.4.2 Open field test

The open field test was performed to determine the activity, exploratory behaviour and non-associated memory. The apparatus was a 60*60*50cm plastic box, and the ground was divided into 9 equal squares by black lines. Mice were placed on the left rear quadrant and left alone to explore the area for 5min in the training session. Then, during the test session, mice were submitted to the same filed at designated time point after CLP or sham surgery. Line crossing times and central square entries were recorded automatically, and a high frequency of these behaviour suggested good motor performance and a low level of anxiety.

### 2.5 Cytokines and oxidative stress quantification

According to the results of behavioral tests, a high dose of β-PAE(1mg/kg) was selected to further determine the potential biological mechanisms underlying the beneficial effects. Mice were euthanized by decapitation at 24h or 7days after surgery. The left cerebral hemisphere was immediately homogenized with a Potter-Elvehjem homogenizer in Tris-HCl buffer, and the right cerebral hemisphere was frozen for further measurement. Expression of TNF-α, IL-1β, IL-6 and IL-10 were measured by ELISA (R&D, Minneapolis, USA) according to the manufacturer’s instructions. ROS and malondialdehyde (MDA) production were detected by commercial available kits (Beyotime, Shanghai, China).

### 2.6 Immunofluorescence assay

Expression of Iba1 and iNOS on microglia in the hippocampus was determined by immunofluorescence assay. The frozen section was prepared and blocked with 3% bovine serum albumin for 1hour. The specimens were incubated with primary antibodies (anti-CD11b Ab and anti-iNOS Ab diluted at 1:100, anti-Iba-1 diluted at 1:200) at 4°C overnight and then stained with the secondary antibodies (CY3-conjugated Goat anti-rat IgG, FITC-conjugated Goat anti-rabbit IgG diluted at 1:400) at 37°C for 1hour. The nucleus was labeled with DAPI (Sigma Aldrich, MO, US). Sections were independently examined by two experienced pathologists using inverted fluorescence microscope (Leica microsystems, Wetzlar, Germany).

### 2.7 Western-blotting

The brain tissue was homogenized in lysis buffer and protein was collected after centrifugation. A nuclear/cytosolic fractionation kit (Sigma Aldrich, St. Louis, MO, USA) was used to extract the nuclear protein. Expression of Sirt1, Nrf2 and HO-1 were determined, and Histone-3 or GAPDH was used as internal control.

### 2.8 TUNEL assay

Terminal deoxynucleotidyl transferase-mediated deoxyuridine triphosphate nick-end labeling (TUNEL) assay (QIA33; Merck Corporation, San Diego, CA) was used to determine the apoptosis in the spleen. Paraffin section was prepared, deparaffinized in xylene and then rehydrated in a graded alcohol series. The specimens were incubated with terminal deoxynucleotidyl transferase and dUTP digoxigenin at 37°C for 1hour. After washing with PBS, the anti-digoxigenin peroxidase solution was added. Then, the sections were colorized with DAB/H_2_O_2_, and counterstained with bisbenzimide. The number of TUNEL-positive cells was counted in 10 high-power field (HPF) and the mean value was calculated.

### 2.9 Survival study

Sixty mice were randomly assigned to sham, CLP, CLP+β-PAE(1mg/kg) or CLP+β-PAE +ML385 group(n = 15 per group). Survival rate was assessed over the seven subsequent days.

### 2.10 Statistical analysis

Data were shown as mean ±standard deviation, and analyzed with Mann-Whitney test or ANOVA with a post-hoc Tukey test as appropriate. Log-rank test was used to determine the significance in survival study. Statistical analysis was performed by using Prism 8.0 (GraphPad Software, San Diego, CA). A p-value of less than 0.05 was considered to be statistically significant.

## 3. Results

### 3.1 β-PAE alleviated the cognitive dysfunction in septic mice

**Fig 1:**
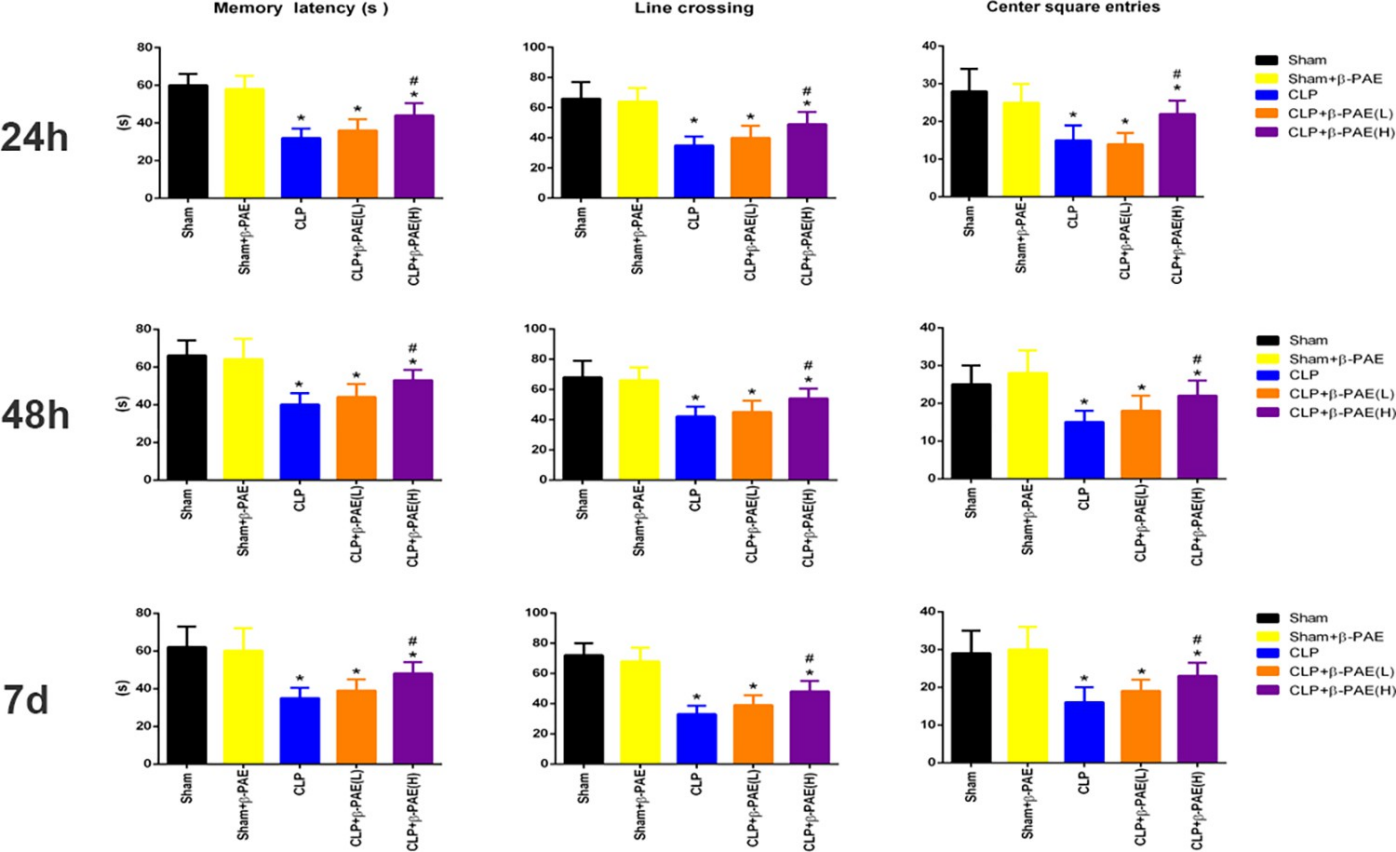
β-PAE improved the cognitive function in SAE mice. Mice were *i*.*c*.*v* injected with a low dose(0.2mg/kg) of β-PAE, high dose(1mg/kg) of β-PAE or saline, respectively. Reagents were all prepared in equal volume of 3μL. Step down inhibitory avoidance test and open field test were performed at 24h, 48h and 7days post-surgery. All presented data are a composite of three independent experiments (n = 6–10 in each group). *P<0.05 vs. Sham control; #P<0.05 vs. CLP control; β-PAE(L), low dose of β-PAE group; β-PAE(H), high dose of β-PAE group.

The behaviour performance was determined at 24h, 48h and 7days after procedures. As shown in Fig 1, sepsis induced significant cognitive impairment at various time points. Septic mice showed a significant decrease in the memory latency time in the test of inhibitory avoidance task. Moreover, SAE mice had a lower frequency of line crossing and center square entries in the open field test. Particularly, a high dose of β-PAE(1mg/kg) significantly improved the learning memory, exploratory activity and motility, as evidenced by an increase in the memory latency time, frequency of line crossing and center square entries.

### 3.2 β-PAE altered the inflammatory response and oxidative stress

**Fig 2:**
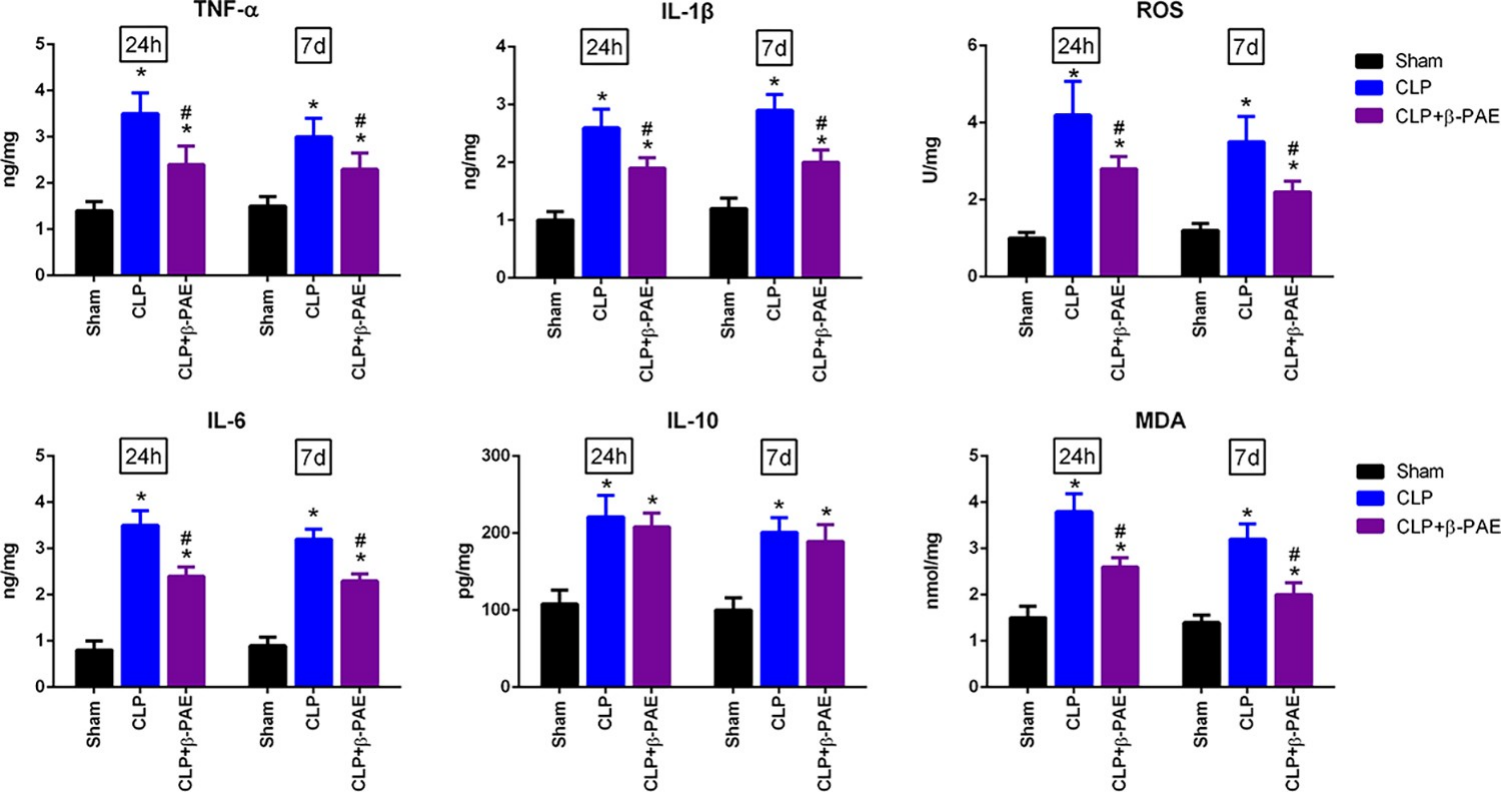
β-PAE suppressed the production of cytokines and oxidative species. Mice were treated with β-PAE(1mg/kg) or saline at the onset of CLP model. Cytokines (TNF-α, IL-1β, IL-6 and IL-10) and oxidative species(ROS and MDA) were determined in the brain homogenate. Data are obtained from three independent experiments (n = 6–8 in each group). *P<0.05 vs. Sham control; #P<0.05 vs. CLP group.

In consistent with the behaviour performance, sepsis led to a substantial neuroinflammation and oxidative stress in the brain. CLP-induced experimental sepsis resulted in a significant upregulation in the expression levels of TNF-α, IL-1β, IL-6, IL-10, ROS and MDA. Administration with β-PAE downregulated the production of pro-inflammatory cytokines and oxidative parameters whereas having no apparent effects on IL-10 expression (Fig 2).

### 3.3 β-PAE reversed the microglia activation and M1 polarization

**Fig 3:**
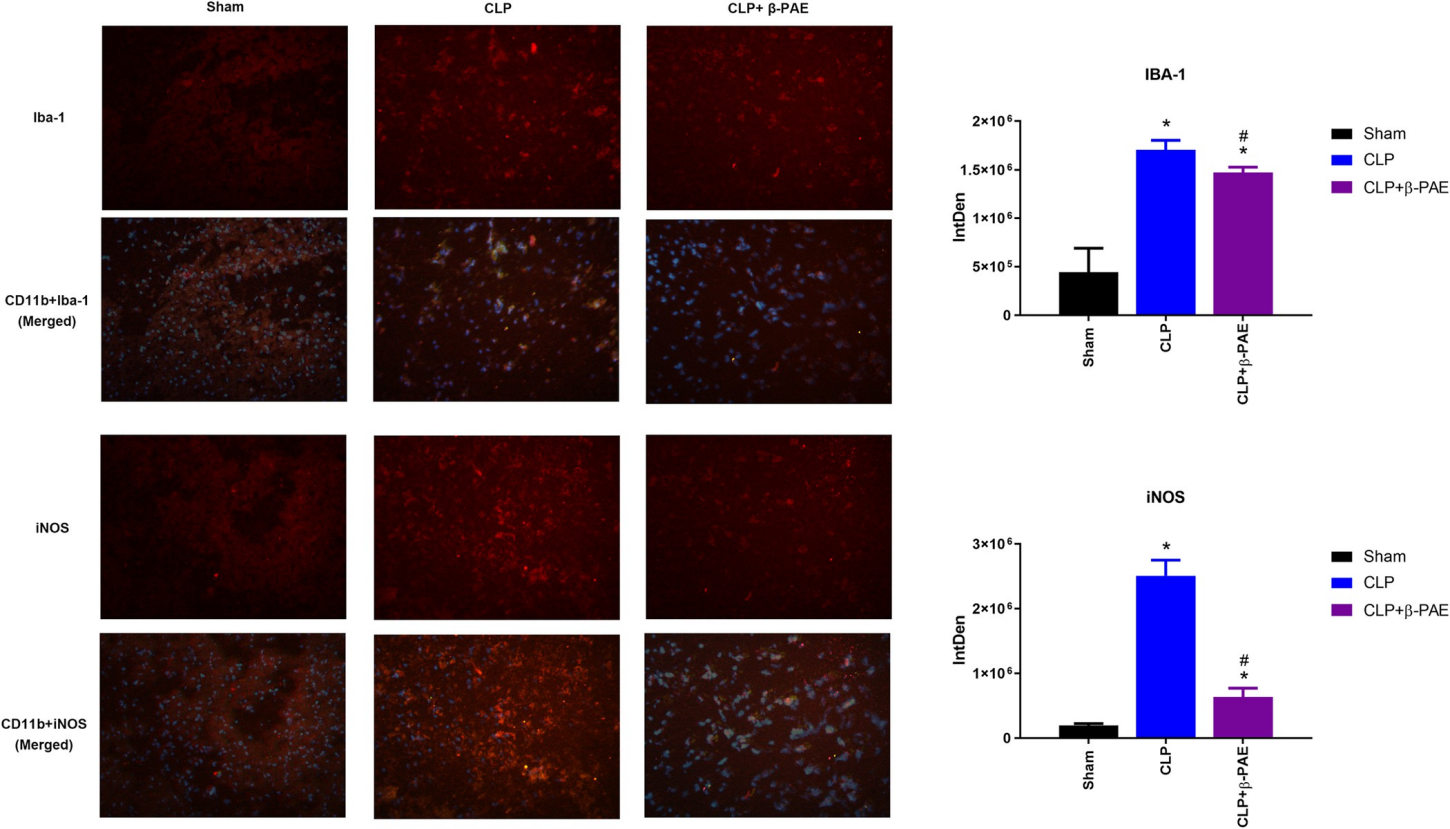
β-PAE suppressed the microglia activation and M1 polarization in SAE. Iba-1 and iNOS expression in hippocampus were determined by immunofluorescence(IF) at 24h post-surgery. Specimens were stained with CD11b(green), Iba-1(red), iNOS(red) or DAPI(blue) respectively. Representative images were merged and shown (1:200). Data are obtained from three independent experiments (n = 4–5 in each group). *P<0.05 vs. Sham control; #P<0.05 vs. CLP group.

As shown in Fig 3, the expression of Iba-1 and iNOS was significantly upregulated at 24h after CLP, implying a substantial activation and pro-inflammatory polarization of microglia occurred in SAE mice. Specially, administration of β-PAE(1mg/kg) downregulated the expression of Iba-1 and iNOS on microglia in the hippocampus.

### 3.4 β-PAE altered the Sirt1/Nrf2/HO-1 signaling pathway and caspase 3 expression

**Fig 4:**
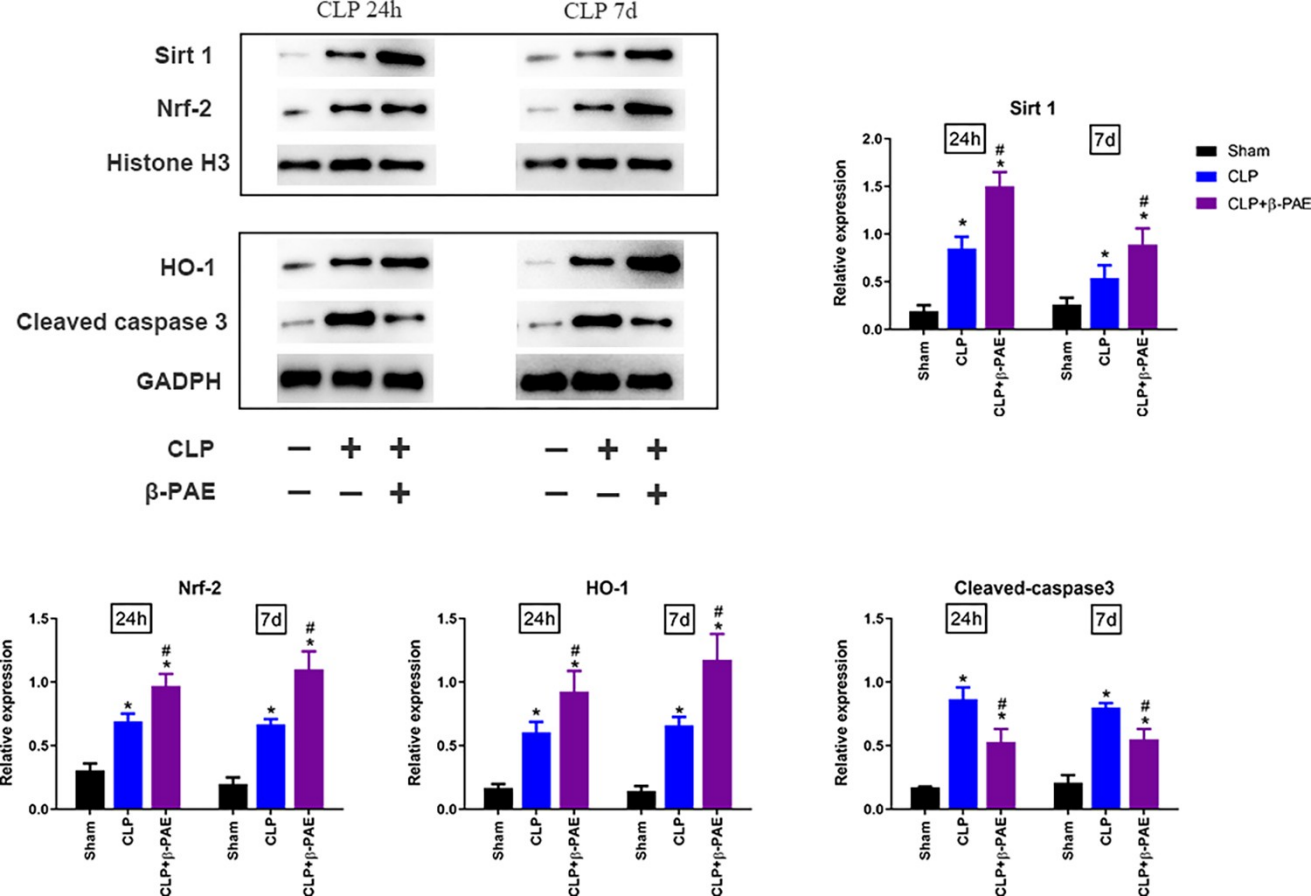
β-PAE altered the Sirt1/Nrf2/HO-1 signaling pathway and caspase 3 activation. Samples of hippocampus were collected and prepared in homogenate at 24h or 7d post-surgery. The concentrations of Sirt1 and Nrf2 in the nuclear protein were measured, and HO-1 and cleaved-caspase 3 were determined in whole protein analysis. Data are obtained from three independent experiments (n = 4–5 in each group). *P<0.05 vs. Sham control; #P<0.05 vs. CLP group.

As shown in Fig 4, septic insult enhanced the nuclear expression of Sirt 1 and Nrf2 in the hippocampus at 24 h post-CLP. Accordingly, HO-1 expression was upregulated in the whole protein analysis. Administration with β-PAE increased the expression of nuclear Sirt1 and Nrf2, and cytoplasmic HO-1. Furthermore, β-PAE inhibited sepsis induced upregulation of cleaved-caspase 3 in the hippocampus, which implied that β-PAE might exert potent anti-apoptotic effects.

### 3.5 β-PAE preserved the peripheral immune function

**Fig 5:**
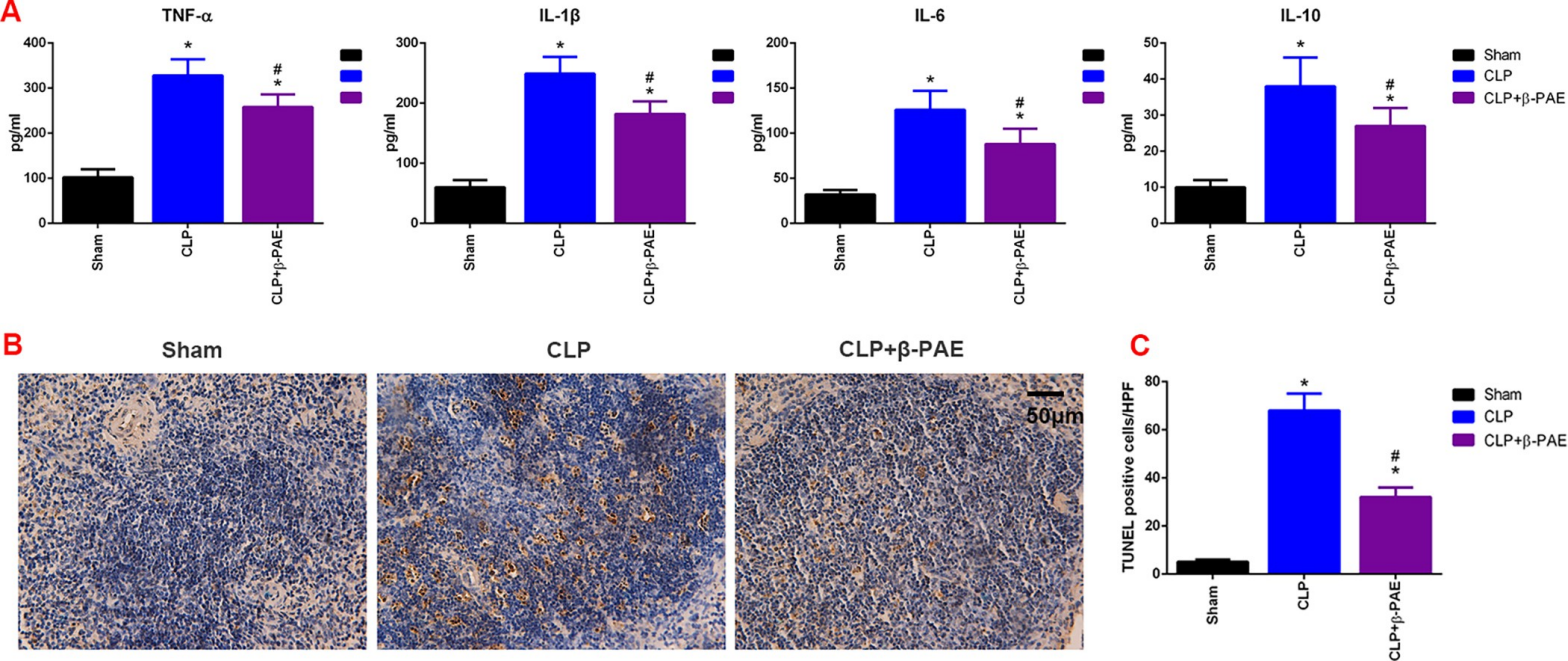
β-PAE downregulated the serum cytokines expression and inhibited lymphocytes apoptosis in the spleen. (A)Expression of TNF-α, IL-1β, IL-6 and IL-10 in serum were determined at 24h post-surgery by ELISA. (B) Representative images of apoptosis in the spleen determined by TUNEL assay (magnification of *400). (C) Summary data of splenocytes apoptosis indicated by mean number of TUNEL positive cells in10 high-power field(HPF). Summary data are obtained from three independent experiments (n = 6–8 in each group). *P<0.05 vs. Sham control; #P<0.05 vs. CLP group.

The serum cytokines expression and spleen lymphocyte apoptosis were determined to assess the peripheral immune function. In parallel with the decreased neuroinflammation, the expression of TNF-α, IL-1β, IL-6 and IL-10 in peripheral blood were downregulated in mice receiving β-PAE. Further, the TUNEL assay revealed a decrease in splenic lymphocyte apoptosis in the β-PAE group. These data suggested that the peripheral immune function was preserved owing to the neuroprotective effects of β-PAE (Fig 5).

### 3.6 Blockade of Nrf2 abolished β-PAE mediated neuroprotective effects

**Fig 6:**
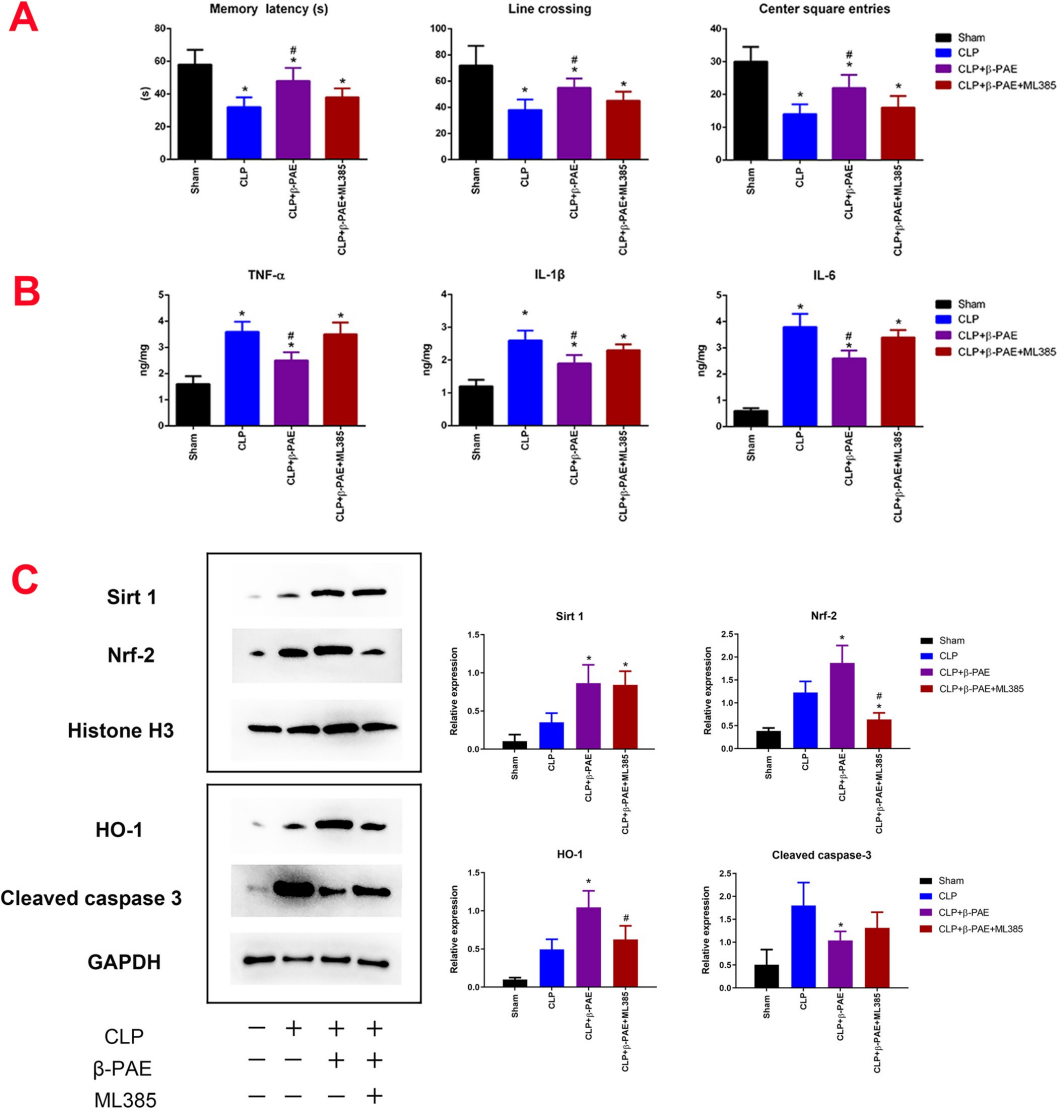
Treatment with ML385 mitigated the protective effects of β-PAE on cognitive function and neuroinflammation. ML385 reversed β-PAE mediated neuroprotective effects as shown in the behavioral test. ML385(1mg/kg in 3μL) was *i*.*c*.*v* injected 2 hours post-CLP. The inhibitory avoidance test and open field test were performed at 24h post-surgery (n = 6–8 in each group). (B) ML385 partially mitigated the anti-inflammatory effects of β-PAE in the brain. TNF-α, IL-1β and IL-6 were measured in brain homogenate by ELISA (n = 6–8 in each group). (C) The protein expression of Sirt1, Nrf2, HO-1 and cleaved-caspase 3 was determined by western-blotting. Samples of hippocampus were collected and prepared in homogenate at 24h after procedure. Summary data are obtained from three independent experiments (n = 4 in each group). *P<0.05 vs.CLP control; #P<0.05 vs. CLP+β-PAE group.

ML385 specifically and directly interacts with Nrf2 protein, blocks the Nrf2 transcriptional activity. As shown in Fig 6, ML385 largely abolished β-PAE mediated upregulation of nuclear Nrf2, cytoplasmic HO-1and cleaved-caspase-3 in the hippocampus. The inhibition of Nrf2 has no apparent effects on the Sirt1 expression. β-PAE-induced downregulation of TNF-α, IL-1β, IL-6 in brain homogenate was mitigated by the administration of ML385. Moreover, ML385 weakened the neuroprotective effects of β-PAE as seen in the inhibitory avoidance task and open filed test. These data suggested that β-PAE mediated anti-inflammation and cognitive protection might be partially through the Nrf2/HO-1 pathway.

### 3.7 β-PAE improved the 7-day survival of SAE mice

**Fig 7:**
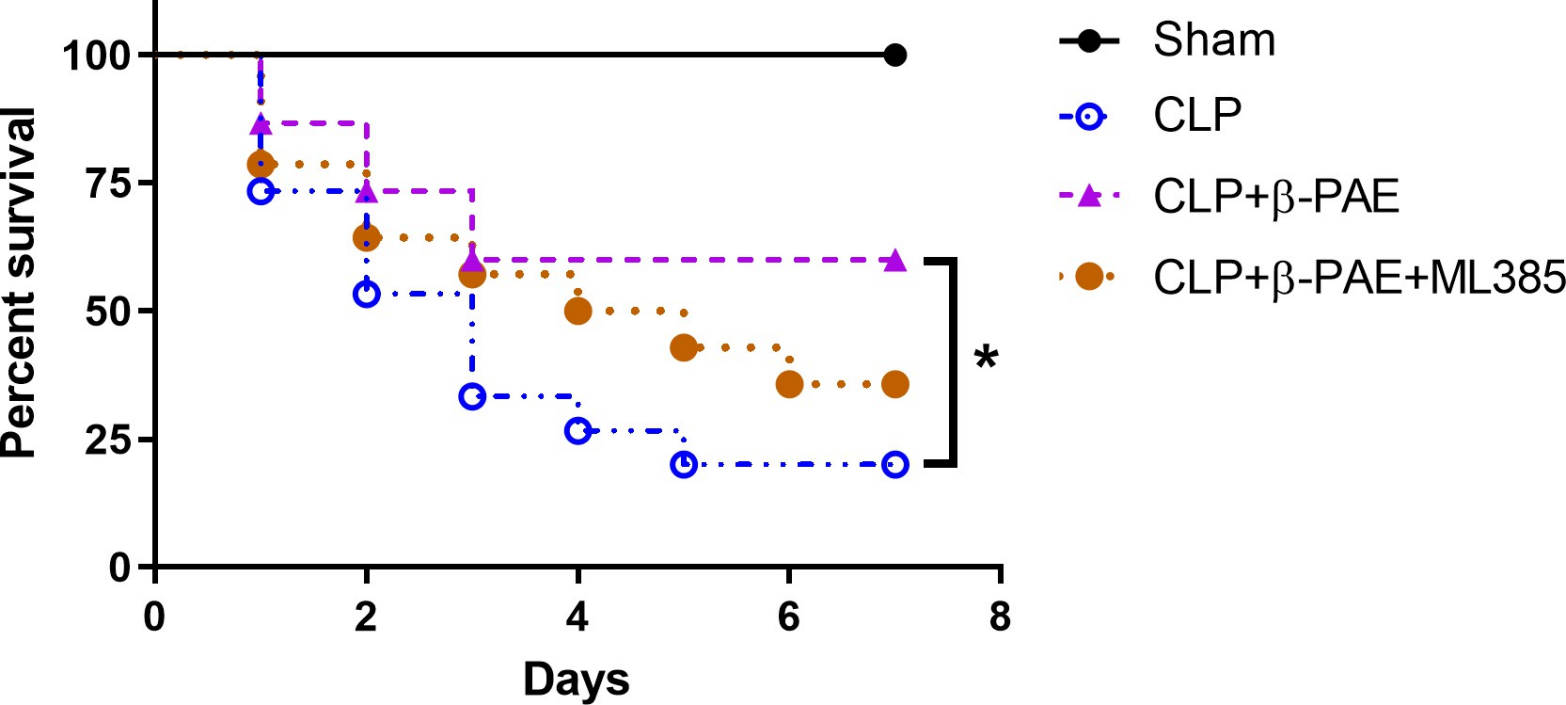
*I*.*c*.*v* treatment with β-PAE improved 7-day survival in septic mice. Mice were *i*.*c*.*v* administrated with β-PAE(1mg/kg) or saline at the onset of CLP model. ML385 was *i*.*c*.*v* injected 2h post-CLP. Data are shown as percentage survival after 7 days. The presented data are a composite of three independent experiments (n = 15 in each group). *P<0.05 *vs*. CLP group.

All sham-operated mice survived in the study period, and the septic mice had a low survival rate of 3/15 (Fig 7). Administration with β-PAE(1mg/kg) effectively boosted the survival rate as compared to the CLP group (3/15 vs. 9/15, p = 0.04), and blockade of Nrf2 by ML385 significantly abolished the protective effects of β-PAE in septic mice.

## 4 Discussion

The central nervous system is particularly vulnerable to the septic insult, mainly mediated by the excessive neuroinflammation and oxidative stress [16]. Despite steady progress has been achieved in the understanding of SAE, the precise mechanisms underlying cerebral dysfunction remains elusive. Currently, the feasible treatment to this complex condition remains inadequate. In the present study, we found that β-PAE successfully reversed the cognitive decline in a mouse model of SAE, which was accompanied by decreased microglia activation, neuroinflammation, oxidative stress and apoptosis. The potential mechanism underpinning β-PAE mediated neuroprotective effects was possibly through the activation of Sirt-1/Nrf2/HO-1 pathway. Hence, β-PAE might serve as a new promising therapeutic agent for treating this ancient disease.

Microglia represent a specialized population of macrophage, which act as the first and main form of active immune defense in the brain. These cells possess phagocytic, migration, proliferation and cytokines secretion capacities [9]. Upon stimulation through the damage- and pathogen- associated molecular patterns or inflammatory mediators, microglia initiate a cascade of cellular response including a characteristic change in cell phenotype [17]. During sepsis, M1 activation of microglia can be aggressive, leading to cytotoxicity and robust inflammation related to the secretion of pro-inflammatory cytokines and oxidative species [18,19]. In this study, we found that M1 polarized microglia predominated in the hippocampus following sepsis, while β-PAE inhibited the M1 polarization and activation. The restoration of phenotypic equilibrium at inflammatory site was likely to facilitate the inhibition of neuroinflammation cascade, which in turn preserved the blood-brain barrier integrity and neuronal cell function. This was inconsistent with the previous studies demonstrating that modulation of microglia activation and polarization seemed to be a feasible approach for SAE treatment [8].

Neuroinflammation is critically involved in the development of SAE, and uncontrolled inflammation is responsible for the dysfunction and massive apoptosis of neuronal cells. The circulating proinflammatory mediators has been shown to enter the CNS by transcellular diffusion, solute carrier proteins, receptor-mediated or adsorptive transcytosis [20]. Meanwhile, pro-inflammatory cytokines can promote the expression of adhesion molecules on endothelial cells, which facilitates the transport of neurotoxic factors and migration of inflammatory cells [21]. As a consequence, the local inflammatory response cascade is initiated and the production of reactive oxygen species is augmented. The uncontrolled neuroinflammation and excessive M1 microglia polarization perpetuated a vicious cycle leading to the neuronal dysfunction or destruction [22]. Moreover, the proinflammatory cytokines including TNF-α, IL-6 and IL-1β in the brain can trigger the behavioral changes, fever and neurological impairment [23,24]. This is in line with the present study identifying that the decrease of pro-inflammatory cytokines expression was associated with the cognitive improvement.

SAE has been identified to be associated with higher mortality in septic patients. One possible reason may be that dysfunction of central nervous system can result in the collapse of neuroendocrine networks, including the hypothalamic-pituitary-adrenal(HPA) axis, the sympathetic and parasympathetic nervous systems [25]. Hence, the peripheral immune disorder observed during SAE can be partially attributed to the overwhelming intracerebral inflammation. Lu *at al* has reported that depletion of peripheral T lymphocytes is independently associated with the development of SAE [26]. The reduction in peripheral T lymphocytes was also observed in the traumatic brain injury, implying that CNS had an essential role in maintaining the functional integrity of host immunity [27]. In this study, we found that sepsis induced a great loss of lymphocytes in the spleen, and notably, *i*.*c*.*v* administration of β-PAE largely prevented the massive apoptosis of peripheral lymphocytes. Theoretically, the preserved host immunity and reduced systemic inflammatory response could facilitate the elimination of invading pathogens, thus improving the survival of septic mice. These data added to the growing body of evidence that mitigation of neuroinflammation appeared to be a promising strategy for the treatment of sepsis associated immune dysfunction.

The anti-inflammatory and anti-oxidative effects of β-PAE have been confirmed in several experimental studies. Zhang *et al* demonstrated that β-PAE exerted a neuroprotective effect on cerebral ischemia/reperfusion(IR) injury in rats through inactivating the TLR4/NF-ƘB signaling pathway [14]. In this study, we found that the mechanisms underlying the protective effects seem to be mediated by the promotion of Nrf2 nuclear translocation and upregulation of Sirt1. It has been reported that activation of Nrf2 can block the M1-stimuli induced production of proinflammatory cytokines and shift the polarization towards a M2-like phenotype [28]. Sirt1 also participated in the maintenance of microglial polarization homeostasis [29], and upregulation of Sirt1 suppressed inflammatory response, oxidative damage and improved cell viability in neurons and microglia co-culture system [30]. In the present study, we found that ML385, a novel and specific Nrf2 inhibitor, largely mitigated β-PAE mediated neuroprotective effects, implying that Sirt1/Nrf2/HO-1 signaling pathways was likely to be involved in the regulation of neuroinflammation in non-redundant ways.

There are several limitations in this study. First, we observed potent anti-inflammatory and anti-oxidative effects of β-PAE in a mouse model of SAE, however, the specific receptor of this novel molecule remains unknown. Second, we treated mice with β-PAE in the initial phase after CLP procedures to determine its potential therapeutic effects, thus the prophylactic treatment and long-term effects on cognitive function remains under-investigation.

## 5 Conclusion

In summary, the results of this study provided evidence for a potential protective role of β-PAE in SAE by showing that β-PAE alleviated microglia activation, neuroinflammation and apoptosis in the brain. β-PAE also suppressed the systemic inflammatory response and reversed the apoptosis of spleen lymphocytes. The protective effect was probably associated with the activation of Sirt1/Nrf2/HO-1 signaling pathways. β-PAE might be a promising therapeutic agent for treating SAE.

## Supporting information

S1 Raw images (PDF)
