# Supplementary material for: β-patchoulene alleviates cognitive dysfunction in a mouse model of sepsis associated encephalopathy by inhibition of microglia activation through Sirt1/Nrf2 signaling pathway
Source: PLoS One. 2023 Jan 6;18(1):e0279964. doi: 10.1371/journal.pone.0279964 (PMC9821490; doi:10.1371/journal.pone.0279964)

Sirt1(nuclear protein in brain homogenate at 24h) see in figure 4.

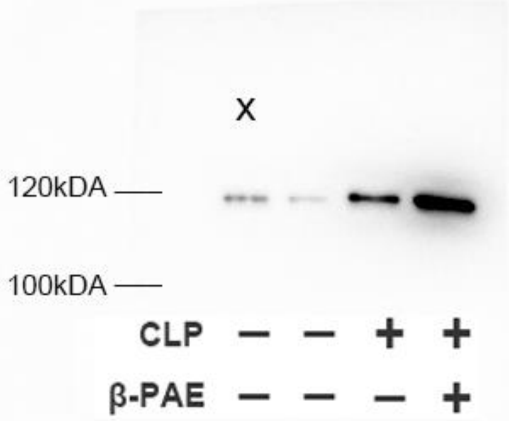

Sirt-1 (nuclear protein in brain homogenate at 7d) see in figure 4.

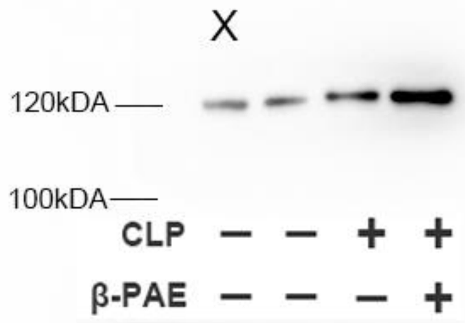

Nrf-2(nuclear protein in brain homogenate at 24h) see in figure 4.

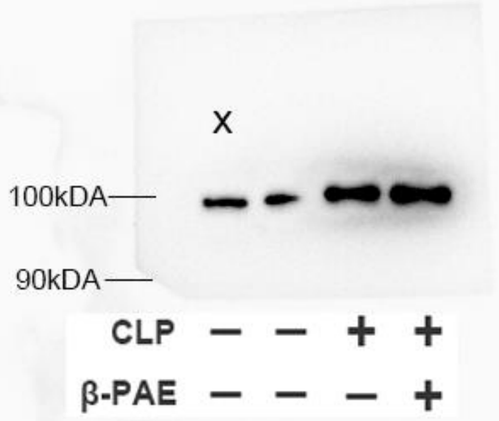

Nrf-2(nuclear protein in brain homogenate at 7d)

see in figure 4.

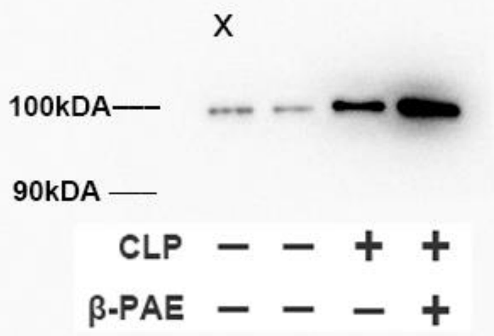

Histone H3(nuclear protein in brain homogenate at 24h)

See in figure 4.

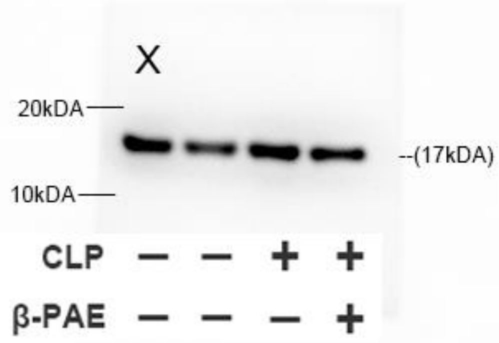

Histone H3 (Nuclear protein in brain homogenate at 7d) see in figure 4.

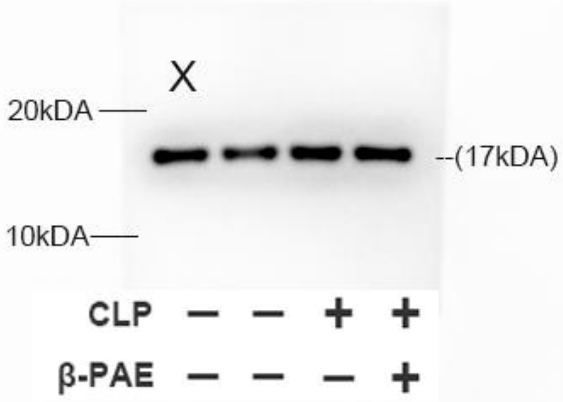

Cleaved-caspase-3 (brain homogenate at 24h)

see in Figure 4

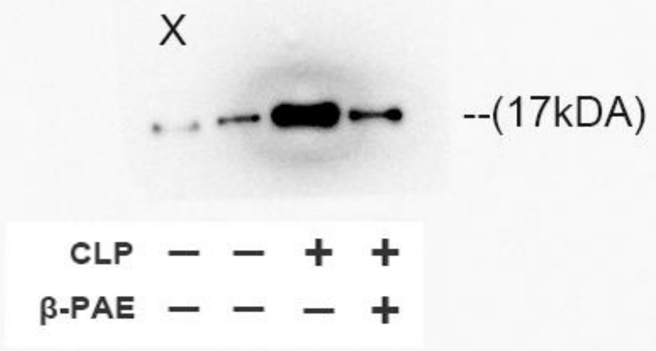

Cleaved-caspase-3 (brain homogenate at 7d)

see in Figure 4.

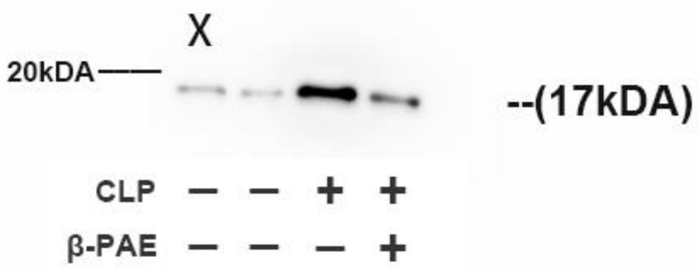

HO-1(brain homogenate at 7d**)**

see in figure 4.

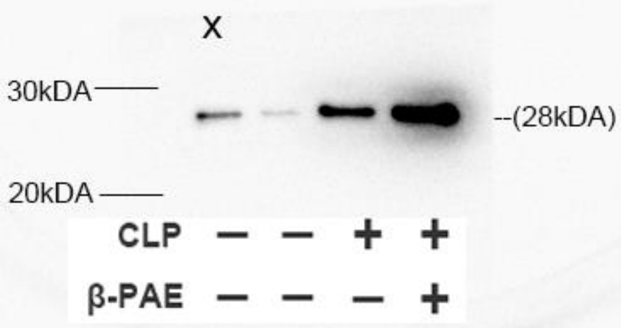

HO-1(brain homogenate at 24h **)**

see in figure 4.

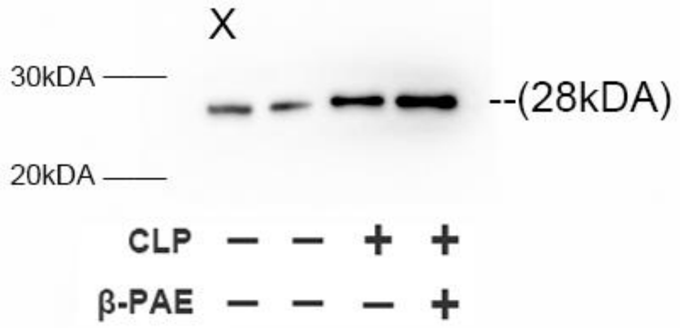

GAPDH(Brain homogenate at 24h)

see in figure 4.

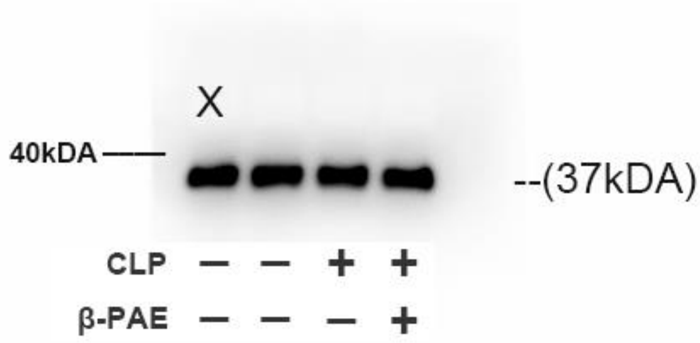

GAPDH (brain homogenate at 7d)

see in figure 4.

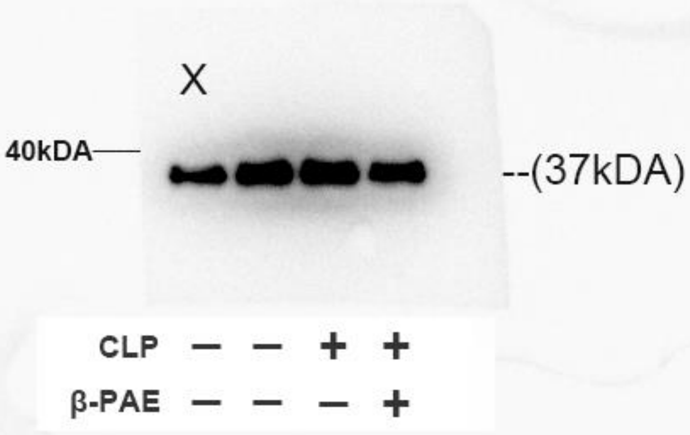

Sirt 1 (nuclear protein in brain homogenate)

see in figure 6.

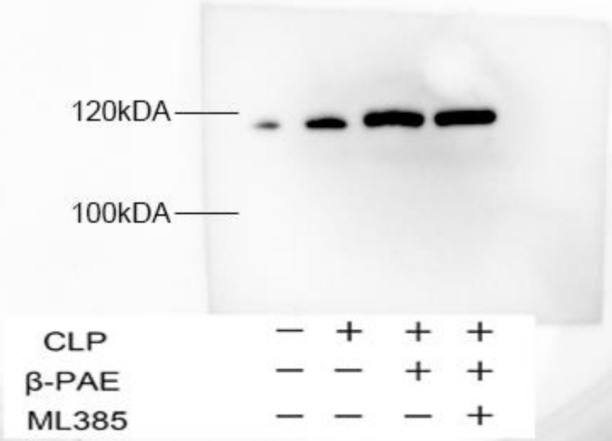

Nrf-2(nuclear protein in brain homogenate)

see in figure 6.

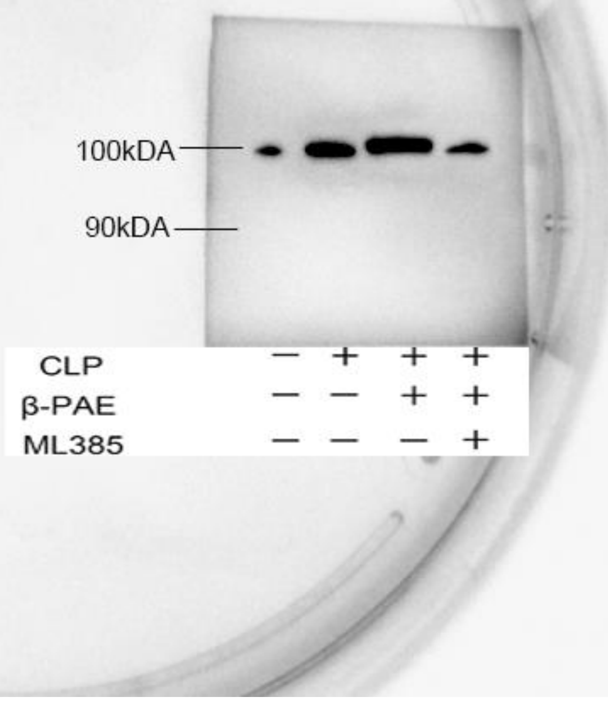

Histone H3 (nuclear protein in brain homogenate**)**

see in figure 6.

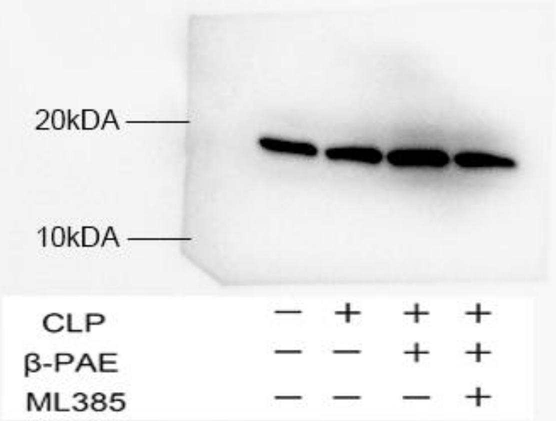

HO-1 (brain homogenate**)**

see in figure 6.

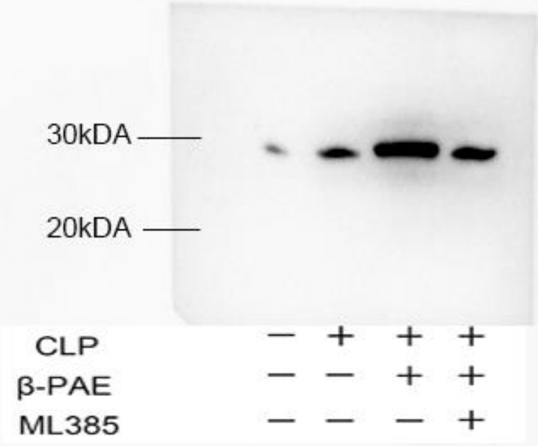

Cleaved-caspase-3(brain homogenate)

see in figure 6.

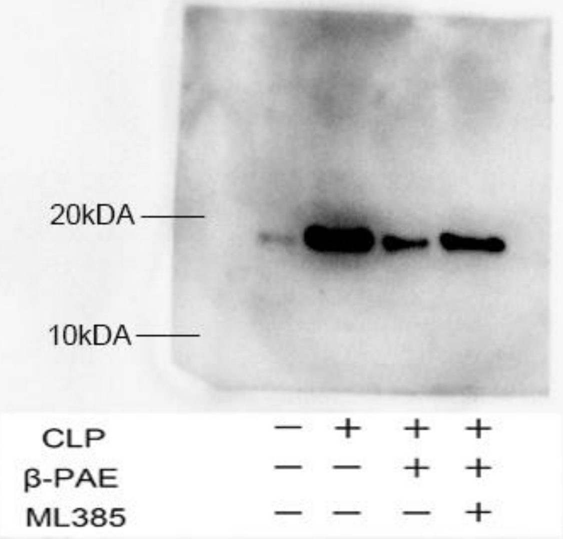

GAPDH(brain homogenate**)**

see in figure 6.

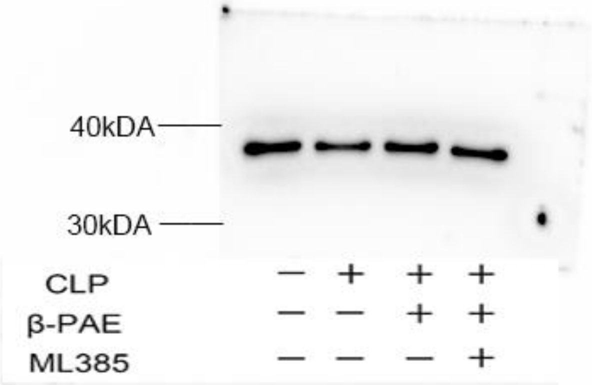

Supplement: S1 Raw images — (PDF) [file pone.0279964.s001.pdf]
